# Pregnancy- and lactation-associated osteoporosis with vertebral fractures: a systematic review

**DOI:** 10.1186/s12891-021-04776-7

**Published:** 2021-11-03

**Authors:** Ying Qian, Lei Wang, Lili Yu, Weimin Huang

**Affiliations:** 1 Endocrinology Department, 960 Hospital of People’s Liberation Army, NO.25 Shifan Road, Jinan, Shandong 250031 People’s Republic of China; 2 Orthopaedic Department, 960 Hospital of People’s Liberation Army, NO.25 Shifan Road, Jinan, Shandong 250031 People’s Republic of China; 3 Medical Information Department, 960 Hospital of People’s Liberation Army, NO.25 Shifan Road, Jinan, Shandong 250031 People’s Republic of China

**Keywords:** Pregnancy, Lactation, Osteoporosis, Vertebral fractures, Systematic review

## Abstract

**Background:**

To review, analyze and characterize the pregnancy and lactation-related osteoporosis (PLO) with vertebral fractures based on the extraction data in the previous studies.

**Methods:**

A comprehensive literature search of electronic databases including the PubMed, Embase and Web of Science was conducted from January 1st,1990 to December 1st, 2020. The enrolled data were pooled to analyze the baseline characteristics, clinical features, risk factors and treatment options.

**Results:**

A total of 65 articles with 338 cases were enrolled for data extraction. The enrolled cases aged from 19 to 47 years, with a mean value of 35.7 years old. The average body mass index (BMI) was 22.2 kg/m^2^ ranged from 16.0 to 39.0 kg/m^2^. Of the 173 cases, 149 cases with vertebral fractures occurred in the first pregnancy, 19 cases in the second pregnancy, four cases in the third pregnancy and one case in the fourth pregnancy. Up to 91.5% of the back pain occurred within the last 3 months of pregnancy and the first 3 months after delivery. The most involved vertebral levels were L2, L1 and T12 accounting for 32.6% of all the fractures. The average fracture numbers were 4.4 levels per patient. The lumbar Z-scores were mostly recorded with a mean value of − 3.2 ranged from − 7.8 to 0.

**Conclusions:**

PLO with vertebral fractures is a rare clinical entity, which is more likely to occur in older and thinner pregnant women. Back pain is the clinical complaint and mostly occurs in the late pregnancy and early lactation periods. Most vertebral fractures appear in the first pregnancy but it can occur in any time of pregnancy. Thoracolumbar region is the mostly involved region. As compared with postmenopausal osteoporotic fractures, PLO usually has multiple levels fractures. Bisphosphonates are the most widely used treatment so far, however, many factors need to be taken into account to decide which drug to choose in PLO and further studies are necessary for clear recommendation in the future.

**Supplementary Information:**

The online version contains supplementary material available at 10.1186/s12891-021-04776-7.

## Background

Pregnancy- and lactation-associated osteoporosis (PLO) is a rare type of osteoporosis that often occurred during the late pregnancy and early lactation [1–4]. Epidemiological data on PLO are limited although previous study has estimated that the prevalence was 4–8 patients per million of population [5]. PLO mainly involves in vertebral body and hip [6–8]. Back pain is one of the most common clinical manifestation and many patients may suffer from vertebral fractures or even kyphosis [9, 10]. PLO carries great physiological and psychological burdens to patients and has negative effects on quality of life and working ability. It was reported that the mean time for the PLO patients returned to work was more than 3 years [11].

Since the first report in 1955 by Nordin, many studies have reported this clinical entity [1–4, 6, 11–23]. Because of the relatively low incidence, most of the studies were case reports and case series, and the clinical features were systematically varied. The patients had experienced back pain differently. Pain can vary from mild to severe and the manifestations of PLO can be present in different trimester of pregnancy. Certain PLO cases occur in the first pregnancy and some occurred in the fourth pregnancy [24]. The patients may have potential risk factors like prior fractures history [25, 26], taking drugs affecting bone metabolism [27–29], smoking and family history of osteoporosis [6, 11, 30]. There is no specific department for PLO. Patients may have attended the Department of Endocrinology, Orthopedics or Obstetrics and Gynecology, however, due to the study limitations and poor awareness, many clinicians have imposing appropriate diagnostic delay and may result in poor prognosis [27].

In order to enhance the knowledge on PLO with vertebral fractures, a systematic review was conducted. We aimed to characterize the clinical manifestation, risk factors, fracture sites and treatment options of PLO based on a data extraction file.

## Methods

### Search terms

A comprehensive literature search of electronic databases including the PubMed, Embase and Web of Science was conducted on December 1st, 2020 to retrieve all articles reporting PLO. The search strategy utilized the following key terms: “Pregnancy OR pregnant OR lactation OR breastfeeding”, “Osteoporosis OR osteoporotic”, “Vertebra OR spine OR spinal OR lumbar OR thoracic OR thoracolumbar”, “Fracture OR fractures”. The search terms were simply contained in the words of the title and abstract of the Pubmed and Embase and in topic terms in the Web of Science. The cases reported in the early literature were seldom diagnosed by using Magnetic resonance imaging (MRI) for vertebral fractures, therefore we only included studies published after January 1st, 1990.

### Inclusion and exclusion criteria

The inclusion and exclusion criteria of the studies were shown in the Table 1.

**Table 1:** Study inclusion and exclusion criteria

Inclusion criteria	Exclusion criteria
All articles on PLO published in English.	1. Basic research.
	2. Editorials, letters or meeting abstract.
	3. Studies that could not found full-text.
	4. Studies that provided too little valuable information to be used for analysis.
	5. Vertebral fractures that had no direct connection with pregnancy or occurred during pregnancy or lactation but having underlying diseases that led to osteoporosis.
	6. Studies on fractures other than vertebral.

*PLO* Pregnancy- and lactation-associated osteoporosis

### Data extraction

Data extraction process was referred to Cochrane Handbook [31]. The retrieved articles were examined and reviewed independently by two researchers. Duplicates were removed automatically by EndNote X8.1 and manually by comparing authors, titles and date of the publications. After the removal of duplicates, title, abstract and full text of articles were screened. Articles reporting the same cohort were also excluded. Then, the supplement search of the references in all the enrolled articles was performed. Data extraction of the selected articles was conducted by two authors using a standard table based on the Cochrane Handbook [31]. The population included was characterized by women affected by PLO and vertebral fractures, and the MRI imaging was used to identify these fractures. For those articles reporting case series, data extractions were performed only in those cases with vertebral fractures. Any disagreements were resolved by a third researcher. In order to unify the standard, the age at onset of symptoms and the height before pregnancy were recorded. Finally, the extracted data were systematically analyzed.

### Quality assessment

Two reviewers independently assessed the quality of the included studies. Disagreements were settled down by discussion among authors. The Joanna Briggs Institute (JBI) critical appraisal checklist for case reports and case series were employed to evaluate the quality of the included studies [32]. There were eight questions in the checklist for case reports and ten questions in the checklist for case series, so the reviewers decided that the studies achieving adequate quality for inclusion should meet a minimum of 50% of the questions requiring a “yes” response.

## Results

### Studies selection process

**Fig. 1:**
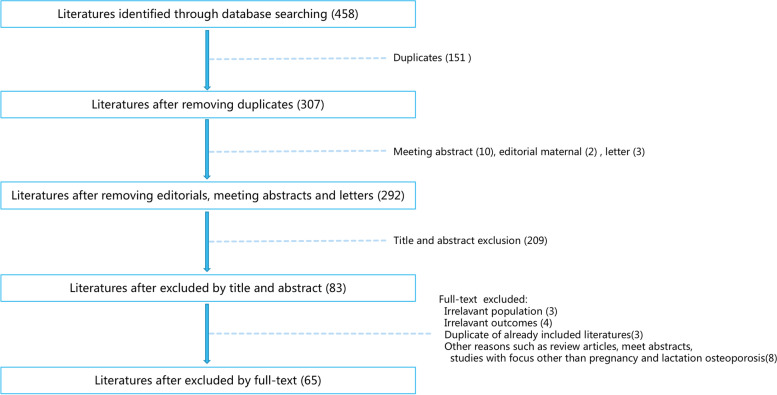
The flow diagram of included and excluded studies

At the initial, 458 articles were retrieved from the database searching, 307 of which remained after duplicates removed. After removing meeting abstract, editorial material and letters, 292 articles were obtained. Of these, 209 were excluded since they did not meet the inclusion criteria. After full text articles assessed for eligibility, another 18 articles were excluded. Finally, 65 articles with 338 cases were enrolled in this systematic review for further data extraction. The flow chart shown in Fig. 1 demonstrates the selection process in detail.

### Study characteristics

Baseline characteristics are showed in Table 2. All the enrolled studies were case report and case series with the case number ranged from 1 to 107 patients. Overall the studies had achieved adequate quality for inclusion (Additional files 1 and 2). Kyvernitakis [1] reported the most cases numbered at 107 based on the German reference center for PLO and Laroche [2] reported the subsequent most cases numbered at 52 based on the French Society of Rheumatology. The number of articles published increased year by year, with 9 articles from 1991 to 2000, 13 articles from 2001 to 2010, and 43 articles from 2011 to 2020. The enrolled studies distributed globally with 34 studies in Europe, 17 studies in Asia, 5 studies in Australia, 5 studies in South America, 3 studies in North America and one study in Africa. Turkey had the highest number of PLO articles which 11 articles recorded, followed by Germany (*n* = 8), Italy (*n* = 7), and South Korea (*n* = 7).

**Table 2:** Characteristics and main findings of the included studies

First author	Published year	Journal	No of Cases ^a^	Race	Age at onset (years, mean ± SD)	Height (cm, mean ± SD)	Weight (kg, mean ± SD)	BMI (kg/m^2^, mean ± SD)
Tuna [3]	2020	Gynecol Endocrinol	9/14		31			21.3
Hardcastle [6]	2019	Osteoporos Int	10	1Moroccan, 9NA	33			23.3
Scott [23]	2019	Osteoporos Int	1	Caucasian	33	162	74	28.2
Ozturk [22]	2019	Gynecol Endocrinol	2		33, 28			27.4, 22.6
Gehlen [11]	2019	Clin Rheumatol	20		33.9			23.5
Zhu [33]	2018	Osteoporos Int	2		29			
Li [20]	2018	Clin Rheumatol	10/12	9Han, 1Manchu	31			21.5
Hong [34]	2018	Clin Endocrinol	32		31.3 ± 2.6			20.3 ± 2.4
Butscheidt [35]	2018	Osteoporos Int	5/7		35			22.8
Taraktas [21]	2018	Turk J Endocrinol Metab	1		22			
Yun [4]	2017	Obstet Gynecol Sci	6		32	164	57	21.1
Kyvernitakis [1]	2017	Osteoporos Int	107		39.5 ± 6.0	165.9 ± 6.3;	63.5 ± 11.1	23.1 ± 3.7
Zhang [36]	2017	Medicine	1		23			21.2
Laroche [2]	2017	Osteoporos Int	52		27			
Ljuin [19]	2017	Taiwan J Obstet Gynecol	1		27	163	45	17.1
Pola [17]	2016	J Biol Regul Homeost Agents	1	Caucasian	33	167	60	21.5
Krishnakumar [37]	2016	J Craniovert Jun Spine	2		27, 31			
Sánchez [18]	2016	Clin Cases Miner Bone Metab	2		35, 33	162, 157		
Grana [9]	2016	Pain Med	1	Caucasian	31			
Gaudio [38]	2016	Clin Cases Miner Bone Metab	1		38	167	54	19.4
Ekim [29]	2016	J Clin Anal Med	1		35	165	54	19.8
Polat [39]	2015	Gynecol Endocrinol	1		23			24.0
Hadgaonkar [40]	2015	Asian Spine J	1		24			
Ozdemir [28]	2015	Osteoporos Int	2		34, 36	168, 162	62, 59	21.9, 22.5
Kovacs [16]	2015	Osteoporos Int	1/2		35	151	46	20.0
Grizzo [26]	2015	Calcif Tissue Int	1	Caucasian	31	165	55	20.2
Zarattini [41]	2014	Clin Cases Miner Bone Metab	1	Caucasian	27	165	63	23.1
Takahashi [42]	2014	Fukushima J Med Sci	1		22	163	60	22.6
Obando [30]	2014	J Clin Endocrinol Metab	1	Caucasian	27	158	53	21.2
Raffaetà [43]	2014	Clin Cases Miner Bone Metab	2		42, 21	167	66	23.7
Ozturk [15]	2014	J Obstet Gynaecol	2		22, 34			
Baldane [44]	2014	Turk Fiz Tip Rehabil Derg	1		35	155	45	18.7
Winarno [45]	2014	Z Geburtsh Neonatol	1		29	158	46	18.4
Terzi [10]	2014	BioMed Res Int	1		32			
Cook [46]	2014	J Bone Miner Res	1	Caucasian	26	161	68	26.2
Scozzari [47]	2014	Acta Medica Mediterranea	1		19			
Lee [48]	2013	J Bone Metab	1		39	156	50	20.5
Bonacker [49]	2013	Arch Orthop Trauma Surg	1		40			
Lwamoto [13]	2012	Ther Clin Risk Manag	1		32	155	57	23.7
Adamidou [50]	2012	Horm-Int J Endocrinol Metab	1	Caucasian	40	158	56	22.4
Choe [51]	2012	J Bone Miner Metab	3		36, 32, 30			20.6, 27.1, 19.4
Stupar [52]	2012	Rheumatol Int	1		30	152	52	22.5
Lee [53]	2011	J Back Musculoskelet Rehabil	1		31	157	50	20.3
Mastaglia [25]	2010	Osteoporos Int	1		20			
Kim [54]	2010	J Korean Neurosurg Soc	1		35	150	42	18.7
Hellmeyer [55]	2010	Gynecol Endocrinol	1		40	171	62	21.2
Tanriover [12]	2009	Spine J	1	Caucasian	23	169	65	22.8
Jang [56]	2009	Rheumatol Int	1		30	163	52	19.6
Ofluoglu [57]	2008	Rheumatol Int	1		30	162	50	19.1
Stumpf [27]	2007	Adv Med Sci	2		32, 41			19.0
Hellmeyer [58]	2007	Exp Clin Endocrinol Diabet	1		28	158	46	18.4
O’Sullivan [59]	2006	Osteoporos Int	10	9Caucasian, 1Fijian	31			22.0
Bayram [60]	2006	Joint Bone Spine	1		37			
Allali [61]	2005	Clin Rheumatol	1		38			
Tran [62]	2002	Intern Med J	3	2Caucasian, 1NA	23, 22, 36	157, 170, 160	47, 48, 47	19.1, 16.6, 18.2
Peris [63]	2002	Clin Exp Rheumatol	5		31	155	54	22.4
Yamaga [64]	2000	Eur J Obstet Gynecol Reprod Biol	1		25			
Gregorio [65]	2000	Nutrition	3	3Caucasian	38, 33, 30	155, 151	56, 47	23.3, 20.5
Anai [66]	1999	J Obstet Gynaecol Res	2		24, 30	161, 155	44, 47	17.0, 19.6
Babbitt [24]	1998	J Clin Densitom	1		46	175	71	23.2
Smith [67]	1995	QJM-Mon J Assoc Physicians	16		28			
Yamamoto [68]	1994	Calcif Tissue Int	5		30	153	57	24.3
Rillo [69]	1994	Clin Rheumatol	1		25			
Blanch [70]	1994	Br J Rheumatol	2	2Caucasian	31, 28			
Reid [71]	1992	Clin Endocrinol	1	Caucasian	31			

*NA* not available

*BMI* body mass index

*PLO* Pregnancy- and lactation-associated osteoporosis

^a^ PLO with vertebral fractures/total population included

### Baseline characteristics of included cases

**Fig. 2:**
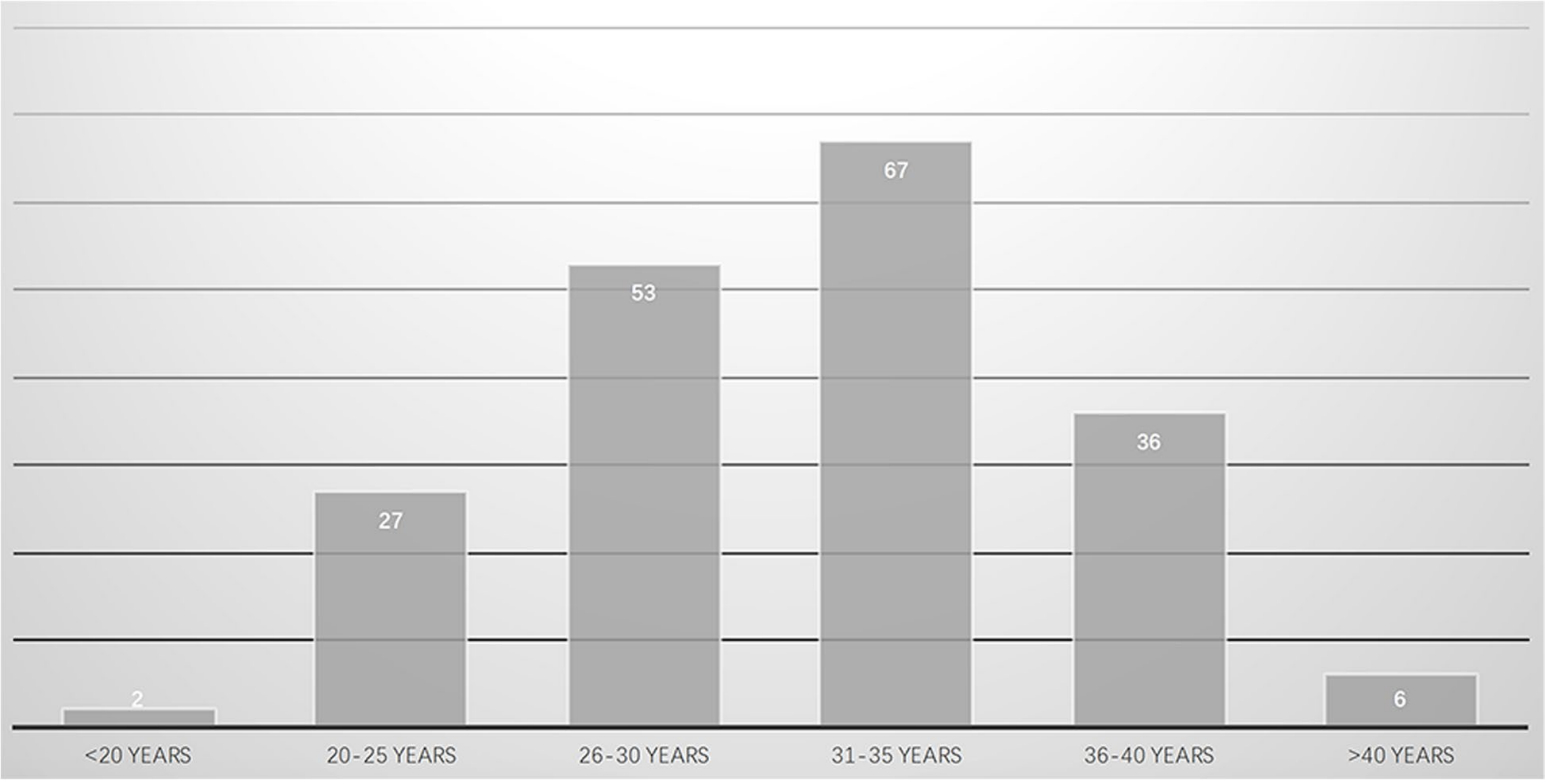
The age distributions of the included population

**Fig. 3:**
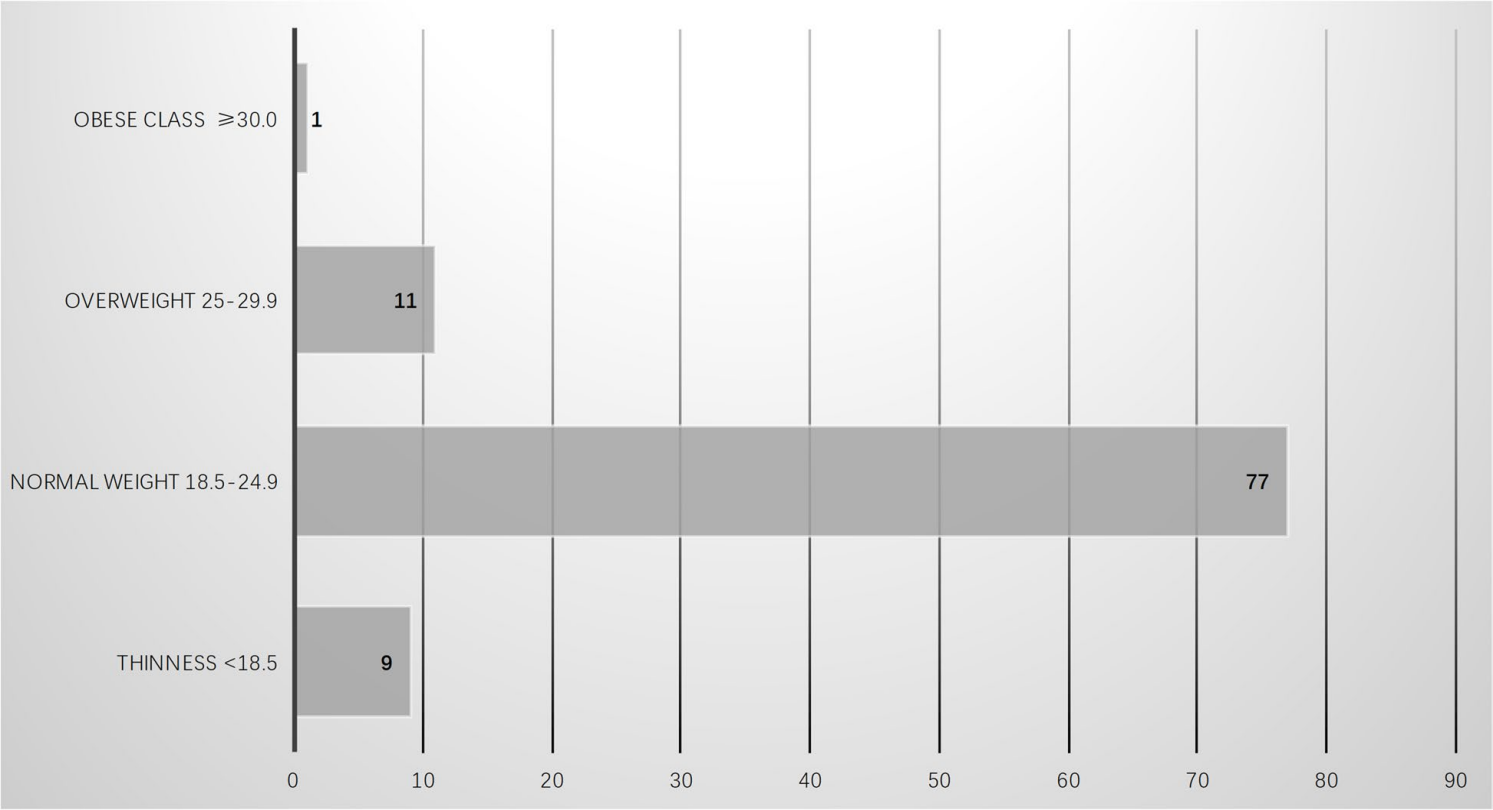
The BMI distributions of the included population

All the included PLO patients aged 19 to 47 years. A total of 191 cases documented the detailed age information with a mean age of 35.7 years. Of the 191 cases, 6 cases over 40 years old accounting for 3.1%, 109 cases over 30 years old accounting for 57.1%, 29 cases under 26 years old accounting for 15.2%. The age distributions are illustrated in Fig. 2. The average height of the included cases is 164.2 cm, ranged from 144 cm to 175 cm. The body mass index (BMI) of 46 studies was calculated and documented with a mean value of 22.2 *kg/m*^*2*^ ranged from 16.0 *kg/m*^*2*^ to 39.0 *kg/m*^*2*^. The BMI distributions of 98 individuals are showed in Fig. 3. The observed data showed that few PLO patients were obese and overweight. Furthermore, race information of 38 cases was documented, which was Caucasians (*n* = 26), Hans (*n* = 9), Manchu (*n* = 1), Fijian (*n* = 1) and Moroccan (*n* = 1).

### Clinical features

A total of 173 cases had the information on number of pregnancy when vertebral fractures occurred, in whom 149 cases were in primiparity, 19 cases were in the second pregnancy, 4 cases were in the third pregnancy and one case was in the fourth pregnancy. There were 108 cases clearly defined feeding manner, with 102 cases breast-feeding accounting for 94.4%. Up to date, not much literature described the delivery way, in which there were vaginal delivery (*n* = 11) and cesarean delivery (*n* = 5).

**Fig. 4:**
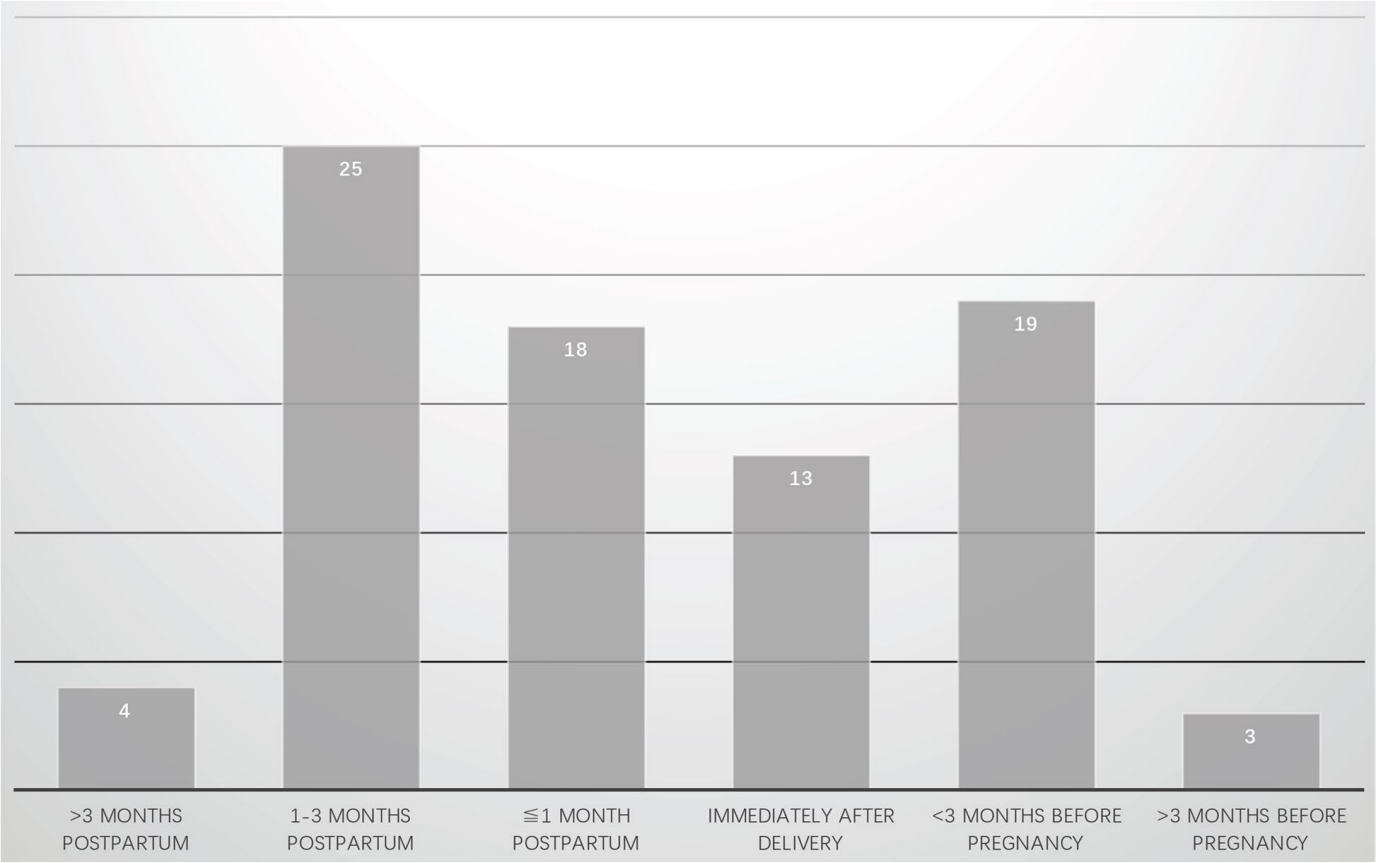
Symptom onset time of the included patients

All the 315 PLO patients with vertebral fractures were symptomized with back pain. The visual analogue score (VAS) were documented in 17 cases, of which all suffered from mild to severe pain and eight cases (47.1%) complained of severe pain. The earliest time of symptom onset was determined at the 5th month pregnancy, while the latest was at 9 months postpartum. Of the 82 cases with definite symptom onset time, 75 cases (91.5%) with back pain occurred during the last 3 months of pregnancy and the first 3 months after delivery. The details of symptom onset time were shown in Fig. 4.

The risk factors associated with PLO were examined such as drug affecting bone metabolism, pre-partum fractures history, family history of osteoporosis, smoking and abnormal menstruation. A total of 59 patients had provided accurate medication history, of which 17 patients (28.8%) had a history of oral anticoagulants such as heparin, low molecular weight heparin (LMWH) and four patients had a corticosteroids history. Of the 68 cases with pre-partum fractures history documented, 17 (25%) cases were suffered from bone fractures before pregnancy. Regarding to family history of osteoporosis, of all the 172 cases with definite documentation, 57 patients (33.1%) had positive family history of osteoporosis. Smoking status was recorded for 111 cases, in which 24 cases (21.6%) were smokers and ex-smokers. There were less menstruation records in the studies, 4 of 25 cases presenting irregular menses.

**Fig. 5:**
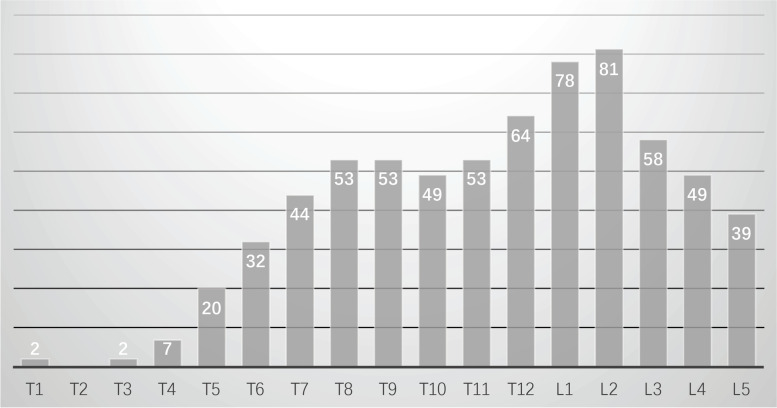
The fractured site of the included population

The studied articles have indicated variable rates of vertebral fractures. Fracture sites were described in 155 cases with 684 vertebral fractures and the average fracture was 4.4 vertebrae per patient. Most cases were suffered from multiple vertebral fractures with only 14 single segment vertebral fractures. As for specific fracture locations, the three most frequently involved vertebral fractures were L2, L1 and T12 (32.6% of all the fractures). The number and site of fractured vertebrae are shown in Fig. 5.

Another important factor is Body mineral density (BMD). The BMD were measured by dual energy x-ray absorptiometry (DXA) in the included studies and the BMD values of the enrolled cases were analyzed. *Z*-scores have been preferable used in the studies as compared to *T*-scores. The lumbar *Z*-scores were recorded in 123 cases in mean value of − 3.2 ranged from − 7.8 to 0, while the hip *Z*-score were recorded in 122 cases with an average of − 2.2 ranged from − 5.5 to 0.9. The lumbar *T*-scores were recorded in 51 cases with an average of − 3.6 ranged from − 6.5 to − 1.3, while the average of the hip T-scores of 47 cases was − 2.5 (ranged from − 6.5 to − 0.2).

### Treatment options

Different therapies that have been used in the management of PLO were documented in 108 cases. These supplementations included Calcium and vitamin D therapy (*n* = 7), bisphosphonates (*n* = 58), teriparatide (*n* = 24), denosumab (*n* = 10), calcitonin (*n* = 6), strontium ranelate (*n* = 2), simple rehabilitation without medication (*n* = 2, with mild symptoms) and vertebroplasty (*n* = 4, with severe symptoms).

## Discussion

The current study demonstrated that PLO is a rare clinical entity and distributed worldwide. To date, although more and more reports are available, the documentation of PLO is still very limited and its mechanism remains unclear. The pooled data revealed PLO is more likely appeared in those pregnant women of advanced maternal age. PLO is an age-related disease [1, 34]. Pregnant women in more than half of the cases were over 30 years.

Similar to postmenopausal osteoporosis, BMI may contribute to increasing risk of PLO. People who are obese or overweight have relatively higher risk of getting PLO.

In general, pregnant women experience calcium loss during the late pregnancy and postpartum lactation. BMD of pregnant women might be associated with pregnancy. In the study of Martina et al. (2010), the prospective changes of BMD with an ultrasonometry measurement in 59 pregnant women were observed. The results showed that BMD was reduced significantly in the second and third trimester of pregnancy [72]. This study indicated that osteopenia is a common condition in pregnant women. However, it is difficult and unethical to measure BMD of pregnant women by X-ray or CT. Contrarily, Lebel et al. (2014) studied the *T*-scores and *Z*-scores of the first 2 days after delivery in 132 pregnant women and found that both scores were within the normal limits regardless of age [73]. These findings indicated that the exact bone metabolism would be more sophisticated in pregnant women.

The pooled data also revealed that PLO may not appear in the first pregnancy. It might be occurred in the second, third or even fourth pregnancy. For patients with multiple pregnancies, PLO might appear in one of them, while other pregnancies were normal [24].

Fracture sites were analyzed in the present study. As compared with other osteoporotic vertebral fractures, PLO had more vertebrae involved. Only a few patients had a single level vertebral fracture. Thoracolumbar region is remained as the most affected area. MRI should be recommended to detect the conditions of thoracic and lumbar vertebrae if cases of missed diagnosis of the fractured vertebrae for the patients with suspected PLO occurred.

Despite its common occurrence, there is no standard clinical guideline for the treatment of PLO. Various kinds of drugs reported in the current reviews have been used in clinical practice for the treatment of PLO, such as bisphosphonates, teriparatide, denosumab and calcitonin. Bisphosphonates are the most used among the drugs. The safety of PLO therapy is always the major concern of clinicians and patients because of its long-term calcium deposits in bones. The use of bisphosphonates may develop adverse effects on both fetus and mother. However, no adverse effects of bisphosphonates on the pregnancy have been reported so far [20, 25, 58]. Calcitonin is more effective for acute pain relief [62, 66]. Denosumab is a human monoclonal antibody and is effective for treatment of osteoporosis by inhibiting the activity of osteoclasts [19]. It has been reported denosumab had achieved satisfactory clinical efficacy when used independently [18] or combined with teriparatide as sequential therapy. Teriparatide is a human parathyroid hormone (PTH) formulation, which helps to regulate calcium metabolism [19, 34, 51]. It has a good prospect for clinic application due to its clinical efficacy and short half-life. However, potential side effect is the risk of bone tumors, which is related to the dosage and duration of treatment [19]. Many factors need to be taken into account to decide which drug to choose in PLO and new drugs and new treatment strategy should be explored in the future [74].

## Conclusion

PLO is a rare clinical type of osteoporosis, which is more likely occur in older and thinner pregnant women. Back pain is a common clinical manifestation during the last 3 months of pregnancy and the first 3 months after delivery. Most PLO occurs in the first pregnancy but it may appear at different stages of pregnancy. Thoracolumbar region is the mostly affected region, however, as compared with postmenopausal osteoporotic fractures, PLO usually has multiple levels fractures. Presently, bisphosphonates are the most widely used treatment for PLO, however, many factors need to be taken into account to decide which drug to choose in PLO and further studies are necessary for clear recommendation in the future.

## Supplementary Information

**Additional file 1.** The references about the included studies.

**Additional file 2.** Quality assessment of the included studies.

**Additional file 3.**

## Data Availability

The datasets used and/or analyzed during the current study are available from the corresponding author on reasonable request.

## Abbreviations

PLO: Pregnancy and lactation-related osteoporosis
MRI: Magnetic resonance imaging
BMI: Body mass index
JBI: Joanna Briggs Institute
VAS: Visual analogue score
LMWH: Low molecular weight heparin
BMD: Body mineral density
DXA: Dual energy x-ray absorptiometry
PTH: Parathyroid hormone
