# Supplementary material for: Pregnancy- and lactation-associated osteoporosis with vertebral fractures: a systematic review
Source: BMC Musculoskelet Disord. 2021 Nov 3;22:926. doi: 10.1186/s12891-021-04776-7 (PMC8567545; doi:10.1186/s12891-021-04776-7)
Supplement: Supplementary file 1 — Additional file 1. The references about the included studies. [file 12891_2021_4776_MOESM1_ESM.docx]

**The references about the included studies.**

1. Tuna F, Akleylek C, Ozdemir H, Demirbag Kabayel D (2020) Risk factors, fractures, and management of pregnancy-associated osteoporosis: a retrospective study of 14 Turkish patients. Gynecological endocrinology : the official journal of the International Society of Gynecological Endocrinology 36:238-242

2. Scott D, Shore-Lorenti C, Ebeling PR (2019) Multiple vertebral compression fractures after sleeve gastrectomy and a subsequent pregnancy: a case report. Osteoporosis Int. 30:2151-2154

3. Ozturk G, Akpinar P, Karamanlioglu AD, Ozkan FU, Aktas I (2019) Pregnancy-related osteoporotic vertebral compression fractures in two patients treated with low-molecular-weight heparin during pregnancy: case reports. 1:46-50

4. Hardcastle SA, Yahya F, Bhalla AK (2019) Pregnancy-associated osteoporosis: a UK case series and literature review. Osteoporosis Int. 30:939-948

5. Gehlen M, Lazarescu AD, Hinz C, Schwarz-Eywill M, Pfeifer M, Balasingam S, Maier A (2019) Long-term outcome of patients with pregnancy and lactation-associated osteoporosis (PLO) with a particular focus on quality of life. Clin. Rheumatol. 38:3575-3583

6. Zhu JJ, Mahendran D, Lee MH, Seah J, Fourlanos S, Varadarajan S, Ghasem-Zadeh A, MacIsaac RJ, Seeman E (2018) Systemic mastocytosis identified in two women developing fragility fractures during lactation. Osteoporosis Int. 29:1671-1674

7. Taraktas A, Unlu Ozkan F, Illeez OG, Kulcu DG, Aktas I (2018) Pregnancy-Associated Osteoporosis: Long-term Follow-up of a Patient with Two Pregnancies. Turk. J. Endocrinol. Metab. 22:50-53

8. Li LJ, Zhang J, Gao P, Lv F, Song YW, Chang XY, Zhao DC, Wang O, Jiang Y, Xing XP, Xia WB, Li M (2018) Clinical characteristics and bisphosphonates treatment of rare pregnancy- and lactation-associated osteoporosis. Clin. Rheumatol. 37:3141-3150

9. Kyvernitakis I, Reuter TC, Hellmeyer L, Hars O, Hadji P (2018) Subsequent fracture risk of women with pregnancy and lactation-associated osteoporosis after a median of 6 years of follow-up. Osteoporosis Int. 29:135-142

10. Hong N, Kim JE, Lee SJ, Kim SH, Rhee Y (2018) Changes in bone mineral density and bone turnover markers during treatment with teriparatide in pregnancy- and lactation-associated osteoporosis. Clin. Endocrinol. 88:652-658

11. Butscheidt S, Delsmann A, Rolvien T, Barvencik F, Al-Bughaili M, Mundlos S, Schinke T, Amling M, Kornak U, Oheim R (2018) Mutational analysis uncovers monogenic bone disorders in women with pregnancy-associated osteoporosis: three novel mutations in LRP5, COL1A1, and COL1A2. Osteoporosis Int. 29:1643-1651

12. Zhang M, Chen P, Li B, Du J, Pan T, Chen J (2017) Approach to the patient with pregnancy and lactation-associated osteoporosis: A case report and a review of the literature. Medicine (Baltimore) 96:e8671

13. Yun KY, Han SE, Kim SC, Joo JK, Lee KS (2017) Pregnancy-related osteoporosis and spinal fractures. Obstetrics & gynecology science 60:133-137

14. Laroche M, Talibart M, Cormier C, Roux C, Guggenbuhl P, Degboe Y (2017) Pregnancy-related fractures: a retrospective study of a French cohort of 52 patients and review of the literature. Osteoporosis Int. 28:3135-3142

15. Krishnakumar R, Kumar AT, Kuzhimattam MJ (2017) Spinal compression fractures due to pregnancy-associated osteoporosis. 1:133-137

16. Ijuin A, Yoshikata H, Asano R, Tsuburai T, Kikuchi R, Sakakibara H (2017) Teriparatide and denosumab treatment for pregnancy and lactation-associated osteoporosis with multiple vertebral fractures: A case study. Taiwan. J. Obstet. Gynecol. 56:863-866

17. Sanchez A, Zanchetta MB, Danilowicz K (2016) Two cases of pregnancy-and lactation-associated osteoporosis successfully treated with denosumab. Clin. Cases Miner. Bone Metab. 13:244-246

18. Pola E, Colangelo D, Nasto LA, Pambianco V, Autore G, Formica VM, Maccauro G (2016) Pregnancy-associated osteoporosis (PAO) with multiple vertebral fragility fractures: diagnosis and treatment in a young primigravid woman. Journal of biological regulators and homeostatic agents 30:153-158

19. Grana E, Invernizzi M, Baricich A, Viscontini GS, Cisari C (2016) A Rare Cause of Back Pain During Pregnancy and Lactation: Management and Treatment. Pain Med. 17:1382-1385

20. Gaudio A, Fiore CE (2016) Successful neridronate therapy in pregnancy-associated osteoporosis. Clin. Cases Miner. Bone Metab. 13:241-243

21. Ekim AA, Eroglu P (2016) Postpartum Osteoporosis and Thoracic Vertebral Fracture in a Patient Treated with Heparin During Pregnancy. J. Clin. Anal. Med. 7:154-157

22. Polat SB, Evranos B, Aydin C, Cuhaci N, Ersoy R, Cakir B (2015) Effective treatment of severe pregnancy and lactation-related osteoporosis with teriparatide: case report and review of the literature. Gynecol. Endocrinol. 31:522-525

23. Ozdemir D, Tam AA, Dirikoc A, Ersoy R, Cakir B (2015) Postpartum osteoporosis and vertebral fractures in two patients treated with enoxaparin during pregnancy. Osteoporosis Int. 26:415-418

24. Kovacs CS, Ralston SH (2015) Presentation and management of osteoporosis presenting in association with pregnancy or lactation. Osteoporosis Int. 26:2223-2241

25. Hadgaonkar S, Shah KC, Bhatt H, Shyam A, Sancheti P (2015) Post Pregnancy Severe Spinal Osteoporosis with Multiple Vertebral Fractures and Kyphoscoliosis in a Multigravida: A Rare Case with Management. Asian spine journal 9:625-628

26. Grizzo FM, da Silva Martins J, Pinheiro MM, Jorgetti V, Carvalho MD, Pelloso SM (2015) Pregnancy and Lactation-Associated Osteoporosis: Bone Histomorphometric Analysis and Response to Treatment with Zoledronic Acid. Calcif Tissue Int 97:421-425

27. Baldane S, Ipekci S, Sahin F, Gul Baldane E, Karabagli P, Kebapcilar L (2015) Subclinical Celiac Disease Presented with Postpartum Low Back Pain Case Report. Turk. Fiz. Tip Rehabil. Derg. 61:175-178

28. Zarattini G, Buffoli P, Isabelli G, Marchese M (2014) Pregnancy-associated osteoporosis with seven vertebral compression fractures, a case treated with strontium ranelate. Clinical cases in mineral and bone metabolism : the official journal of the Italian Society of Osteoporosis, Mineral Metabolism, and Skeletal Diseases 11:139-141

29. Winarno AS, Kyvernitakis I, Hadji P (2014) Successful Treatment of 1-34 Parathyroid Hormone (PTH) after Failure of Bisphosphonate Therapy in a Complex Case of Pregnancy Associated Osteoporosis and Multiple Fractures. Z. Geburtsh. Neonat. 218:171-173

30. Terzi R, Terzi H, Ozer T, Kale A (2014) A Rare Cause of Postpartum Low Back Pain: Pregnancy- and Lactation-Associated Osteoporosis. Biomed Res. Int.:3

31. Takahashi N, Arai I, Kayama S, Ichiji K, Fukuda H, Handa J, Konno S (2014) Four-year follow-up of pregnancy-associated osteoporosis: a case report. Fukushima journal of medical science 60:175-180

32. Scozzari F, Aronica GL, Seidita A, Taormina G, di Stefano L, D'Alcamo A, Carta M, Adragna F, Nugara G, Enna C, Mansueto P (2014) OSTEOPOROSIS IN PREGNANCY: A CASE REPORT AND REVIEW OF THE LITERATURE. Acta Medica Mediterr. 30:115-120

33. Raffaeta G, Mazzantini M, Menconi A, Bottai V, Falossi F, Celauro I, Guido G (2014) Osteoporosis with vertebral fractures associated with pregnancy: two case reports. 1:139-141

34. Ozturk C, Atamaz FC, Akkurt H, Akkoc Y (2014) Pregnancy-associated osteoporosis presenting severe vertebral fractures. 1:288-292

35. Cook FJ, Mumm S, Whyte MP, Wenkert D (2014) Pregnancy-associated osteoporosis with a heterozygous deactivating LDL receptor-related protein 5 (LRP5) mutation and a homozygous methylenetetrahydrofolate reductase (MTHFR) polymorphism. Journal of bone and mineral research : the official journal of the American Society for Bone and Mineral Research 29:922-928

36. Choe EY, Song JE, Park KH, Seok H, Lee EJ, Lim SK, Rhee Y (2014) Effect of teriparatide on pregnancy and lactation-associated osteoporosis with multiple vertebral fractures. 1:596-601

37. Campos-Obando N, Oei L, Hoefsloot LH, Kiewiet RM, Klaver CC, Simon ME, Zillikens MC (2014) Osteoporotic vertebral fractures during pregnancy: be aware of a potential underlying genetic cause. The Journal of clinical endocrinology and metabolism 99:1107-1111

38. Bonacker J, Janousek M, Krober M (2014) Pregnancy-associated osteoporosis with eight fractures in the vertebral column treated with kyphoplasty and bracing: a case report. 1:173-179

39. Lee SH, Hong MK, Park SW, Park HM, Kim J, Ahn J (2013) A case of teriparatide on pregnancy-induced osteoporosis. Journal of bone metabolism 20:111-114

40. Vujasinovic Stupar N, Pejnovic N, Markovic L, Zlatanovic M (2012) Pregnancy-associated spinal osteoporosis treated with bisphosphonates: long-term follow-up of maternal and infants outcome. Rheumatol. Int. 32:819-823

41. Lampropoulou-Adamidou K, Trovas G, Stathopoulos IP, Papaioannou NA (2012) Case Report: Teriparatide treatment in a case of severe pregnancy -and lactation- associated osteoporosis. Horm.-Int. J. Endocrinol. Metab. 11:495-500

42. Iwamoto J, Sato Y, Uzawa M, Matsumoto H (2012) Five-year follow-up of a woman with pregnancy and lactation-associated osteoporosis and vertebral fractures. Therap. Clin. Risk Manag. 8:195-199

43. Lee JH, Lee SH (2011) Multiple vertebral compression fractures associated with post partum osteoporosis. J. Back Musculoskelet. Rehabil. 24:117-121

44. Mastaglia SR, Watman NP, Oliveri B (2010) Intravenous bisphosphonate treatment and pregnancy: its effects on mother and infant bone health. Osteoporosis Int. 21:1959-1962

45. Kim HW, Song JW, Kwon A, Kim IH (2010) Percutaneous Vertebroplasty for Pregnancy-Associated Osteoporotic Vertebral Compression Fractures. Journal of Korean Neurosurgical Society 47:399-402

46. Hellmeyer L, Boekhoff J, Hadji P (2010) Treatment with teriparatide in a patient with pregnancy-associated osteoporosis. Gynecol. Endocrinol. 26:725-728

47. Tanriover MD, Oz SG, Sozen T, Kilicarslan A, Guven GS (2009) Pregnancy- and lactation-associated osteoporosis with severe vertebral deformities: can strontium ranelate be a new alternative for the treatment? The spine journal : official journal of the North American Spine Society 9:e20-24

48. Jang JY, Lee JG, Jeong IK, Ahn KJ, Chung HY, Yang HI, Lee SH (2009) A case of post-pregnancy osteoporosis combined with ankylosing spondylitis. Rheumatol. Int. 29:1359-1362

49. Ofluoglu O, Ofluoglu D (2008) A case report: pregnancy-induced severe osteoporosis with eight vertebral fractures. Rheumatol. Int. 29:197-201

50. Stumpf UC, Kurth AA, Windolf J, Fassbender WJ (2007) Pregnancy-associated osteoporosis: an underestimated and underdiagnosed severe disease. A review of two cases in short- and long-term follow-up. Adv. Med. Sci. 52:94-97

51. Hellmeyer L, Kuehnert M, Ziller V, Schmidt S, Hadji P (2007) The use of i. v. bisphosphonate in pregnancy-associated osteoporosis - Case study. Exp. Clin. Endocrinol. Diabet. 115:139-142

52. O'Sullivan SM, Grey AB, Singh R, Reid IR (2006) Bisphosphonates in pregnancy and lactation-associated osteoporosis. Osteoporosis Int. 17:1008-1012

53. Bayram S, Ozturk C, Sivrioglu K, Aydinli U, Kucukoglu S (2006) Kyphoplasty for pregnancy-associated osteoporotic vertebral fractures. Joint bone spine 73:564-566

54. Allali F, Guedirra N, Hajjaj-Hassouni N (2005) A case of post-pregnancy osteoporosis-related spinal fractures in association with ankylosing spondylitis. Clin. Rheumatol. 24:435-436

55. Tran HA, Petrovsky N (2002) Pregnancy-associated osteoporosis with hypercalcaemia. Intern. Med. J. 32:481-485

56. Peris P, Guanabens N, Monegal A, Pons F, de Osaba MJM, Ros I, Munoz-Gomez J (2002) Pregnancy associated osteoporosis: The familial effect. Clin. Exp. Rheumatol. 20:697-700

57. Yamaga A, Taga M, Takahashi T, Shirai T (2000) A case of postpregnancy osteoporosis. Eur. J. Obstet. Gynecol. Reprod. Biol. 88:107-109

58. Di Gregorio S, Danilowicz K, Rubin Z, Mautalen C (2000) Osteoporosis with vertebral fractures associated with pregnancy and lactation. Nutrition 16:1052-1055

59. Anai T, Tomiyasu T, Arima K, Miyakawa I (1999) Pregnancy-associated osteoporosis with elevated levels of circulating parathyroid hormone-related protein: a report of two cases. The journal of obstetrics and gynaecology research 25:63-67

60. Babbitt AM (1998) Post-pregnancy osteoporosis (PPO) - A case study. J. Clin. Densitom. 1:269-273

61. Smith R, Athanasou NA, Ostlere SJ, Vipond SE (1995) Pregnancy-associated osteoporosis. QJM-Mon. J. Assoc. Physicians 88:865-878

62. Yamamoto N, Takahashi HE, Tanizawa T, Kawashima T, Endo N (1994) BONE-MINERAL DENSITY AND BONE HISTOMORPHOMETRIC ASSESSMENTS OF POSTPREGNANCY OSTEOPOROSIS - A REPORT OF 5 PATIENTS. Calcif. Tissue Int. 54:20-25

63. Rillo OL, Di Stefano CA, Bermudez J, Maldonado Cocco JA (1994) Idiopathic osteoporosis during pregnancy. Clin Rheumatol 13:299-304

64. Blanch J, Pacifici R, Chines A (1994) Pregnancy-associated osteoporosis: report of two cases with long-term bone density follow-up. Br J Rheumatol 33:269-272

65. Reid IR, Wattie DJ, Evans MC, Budayr AA (1992) POST-PREGNANCY OSTEOPOROSIS ASSOCIATED WITH HYPERCALCEMIA. Clin. Endocrinol. 37:298-303
