# Supplementary material for: Pregnancy- and lactation-associated osteoporosis with vertebral fractures: a systematic review
Source: BMC Musculoskelet Disord. 2021 Nov 3;22:926. doi: 10.1186/s12891-021-04776-7 (PMC8567545; doi:10.1186/s12891-021-04776-7)
Supplement: Supplementary file 2 — Additional file 2. Quality assessment of the included studies. [file 12891_2021_4776_MOESM2_ESM.docx]

| Table 3 Quality assessment of the included studies | | | | | | | | | | | | | | |
| --- | --- | --- | --- | --- | --- | --- | --- | --- | --- | --- | --- | --- | --- | --- |
| Number | First author | Location | Published year | Q1 | Q2 | Q3 | Q4 | Q5 | Q6 | Q7 | Q8 | Q9 | Q10 | Quality assessment^a^ |
| 1 | Tuna | Turkey | 2020 | 1 | 1 | 1 | 0 | 1 | 1 | 1 | 1 | 1 | 1 | 9 |
| 2 | Hardcastle | UK | 2019 | 1 | 1 | 1 | 1 | 1 | 1 | 0 | 1 |  |  | 7 |
| 3 | Scott | Australia | 2019 | 1 | 1 | 1 | 1 | 1 | 1 | 1 | 1 |  |  | 8 |
| 4 | Ozturk | Turkey | 2019 | 1 | 1 | 1 | 1 | 1 | 1 | 0 | 1 |  |  | 7 |
| 5 | Gehlen | Germany | 2019 | 1 | 1 | 1 | 0 | 1 | 1 | 1 | 1 | 1 | 1 | 9 |
| 6 | Zhu | Australia | 2018 | 1 | 1 | 1 | 0 | 1 | 1 | 0 | 1 |  |  | 6 |
| 7 | Li | China | 2018 | 1 | 1 | 1 | 0 | 1 | 1 | 1 | 1 | 1 | 1 | 9 |
| 8 | Hong | Korea | 2018 | 1 | 1 | 1 | 0 | 0 | 1 | 1 | 1 | 1 | 1 | 8 |
| 9 | Butscheidt | Germany | 2018 | 1 | 1 | 1 | 1 | 1 | 1 | 0 | 0 | 1 | 1 | 8 |
| 10 | Taraktas | Italy | 2018 | 1 | 1 | 1 | 1 | 1 | 1 | 1 | 1 |  |  | 8 |
| 11 | Yun | Korea | 2017 | 1 | 1 | 1 | 1 | 1 | 1 | 1 | 1 |  |  | 8 |
| 12 | Kyvernitakis | Germany | 2017 | 1 | 1 | 1 | 0 | 1 | 1 | 1 | 1 | 1 | 1 | 9 |
| 13 | Zhang | China | 2017 | 1 | 1 | 1 | 1 | 1 | 1 | 0 | 1 |  |  | 7 |
| 14 | Laroche | France | 2017 | 0 | 1 | 1 | 0 | 1 | 1 | 1 | 1 | 1 | 1 | 8 |
| 15 | Ljuin | Japan | 2017 | 1 | 1 | 1 | 1 | 1 | 1 | 0 | 1 |  |  | 7 |
| 16 | Pola | Italy | 2016 | 1 | 1 | 1 | 1 | 1 | 1 | 1 | 1 |  |  | 8 |
| 17 | Krishnakumar | India | 2016 | 1 | 0 | 1 | 1 | 1 | 1 | 0 | 1 |  |  | 6 |
| 18 | Sánchez | Argentina | 2016 | 1 | 1 | 1 | 1 | 1 | 1 | 1 | 1 |  |  | 8 |
| 19 | Grana | Italy | 2016 | 1 | 1 | 1 | 1 | 1 | 1 | 1 | 1 |  |  | 8 |
| 20 | Gaudio | Italy | 2016 | 1 | 1 | 1 | 1 | 1 | 1 | 0 | 1 |  |  | 7 |
| 21 | Ekim | Turkey | 2016 | 1 | 1 | 1 | 1 | 1 | 1 | 0 | 1 |  |  | 7 |
| 22 | Polat | Turkey | 2015 | 1 | 1 | 1 | 1 | 1 | 1 | 0 | 1 |  |  | 7 |
| 23 | Hadgaonkar | India | 2015 | 1 | 1 | 1 | 1 | 1 | 1 | 0 | 1 |  |  | 7 |
| 24 | Ozdemir | Turkey | 2015 | 1 | 1 | 1 | 1 | 1 | 1 | 0 | 1 |  |  | 7 |
| 25 | Kovacs | Canada | 2015 | 1 | 1 | 1 | 1 | 1 | 1 | 0 | 1 |  |  | 7 |
| 26 | Grizzo | Brazil | 2015 | 1 | 1 | 1 | 1 | 1 | 1 | 1 | 1 |  |  | 8 |
| 27 | Zarattini | Italy | 2014 | 1 | 1 | 1 | 1 | 1 | 1 | 0 | 1 |  |  | 7 |
| 28 | Takahashi | Japan | 2014 | 1 | 1 | 1 | 1 | 1 | 1 | 0 | 1 |  |  | 7 |
| 29 | Obando | Netherlands | 2014 | 1 | 1 | 1 | 1 | 1 | 1 | 0 | 1 |  |  | 7 |
| 30 | Raffaetà | Italy | 2014 | 1 | 1 | 1 | 1 | 1 | 1 | 0 | 1 |  |  | 7 |
| 31 | Ozturk | Turkey | 2014 | 1 | 1 | 1 | 1 | 1 | 1 | 0 | 1 |  |  | 7 |
| 32 | Baldane | Turkey | 2014 | 1 | 1 | 1 | 1 | 1 | 1 | 0 | 1 |  |  | 7 |
| 33 | Winarno | Germany | 2014 | 1 | 1 | 1 | 1 | 1 | 1 | 0 | 1 |  |  | 7 |
| 34 | Terzi | Turkey | 2014 | 1 | 1 | 1 | 1 | 1 | 1 | 0 | 1 |  |  | 7 |
| 35 | Cook | USA | 2014 | 1 | 1 | 1 | 1 | 1 | 1 | 1 | 1 |  |  | 8 |
| 36 | Scozzari | Italy | 2014 | 1 | 1 | 1 | 1 | 1 | 1 | 0 | 1 |  |  | 7 |
| 37 | Lee | Korea | 2013 | 1 | 1 | 1 | 1 | 1 | 1 | 0 | 1 |  |  | 7 |
| 38 | Bonacker | Germany | 2013 | 1 | 1 | 1 | 1 | 1 | 1 | 0 | 1 |  |  | 7 |
| 39 | Lwamoto | Japan | 2012 | 1 | 1 | 1 | 1 | 1 | 1 | 1 | 1 |  |  | 8 |
| 40 | Adamidou | Greece | 2012 | 1 | 1 | 1 | 1 | 1 | 1 | 0 | 1 |  |  | 7 |
| 41 | Choe | Korea | 2012 | 1 | 1 | 1 | 1 | 1 | 1 | 0 | 1 |  |  | 7 |
| 42 | Stupar | Serbia | 2012 | 1 | 1 | 1 | 1 | 1 | 1 | 0 | 1 |  |  | 7 |
| 43 | Lee | Korea | 2011 | 1 | 1 | 1 | 1 | 1 | 1 | 0 | 1 |  |  | 7 |
| 44 | Mastaglia | Argentina | 2010 | 1 | 1 | 1 | 1 | 1 | 1 | 0 | 1 |  |  | 7 |
| 45 | Kim | Korea | 2010 | 1 | 1 | 1 | 1 | 1 | 1 | 0 | 1 |  |  | 7 |
| 46 | Hellmeyer | Germany | 2010 | 1 | 1 | 1 | 1 | 1 | 1 | 0 | 1 |  |  | 7 |
| 47 | Tanriover | Turkey | 2009 | 1 | 1 | 1 | 1 | 1 | 1 | 0 | 1 |  |  | 7 |
| 48 | Jang | Korea | 2009 | 1 | 1 | 1 | 1 | 1 | 1 | 0 | 1 |  |  | 7 |
| 49 | Ofluoglu | Turkey | 2008 | 1 | 1 | 1 | 1 | 1 | 0 | 0 | 1 |  |  | 6 |
| 50 | Stumpf | Germany | 2007 | 1 | 1 | 1 | 1 | 1 | 1 | 0 | 1 |  |  | 7 |
| 51 | Hellmeyer | Germany | 2007 | 1 | 1 | 1 | 1 | 1 | 1 | 0 | 1 |  |  | 7 |
| 52 | O’Sullivan | New Zealand | 2006 | 0 | 1 | 1 | 0 | 1 | 1 | 1 | 1 | 1 | 1 | 8 |
| 53 | Bayram | Turkey | 2006 | 1 | 1 | 1 | 1 | 1 | 1 | 0 | 1 |  |  | 7 |
| 54 | Allali | Morocco | 2005 | 1 | 0 | 1 | 1 | 1 | 1 | 0 | 1 |  |  | 6 |
| 55 | Tran | Australia | 2002 | 1 | 1 | 1 | 1 | 1 | 1 | 0 | 1 |  |  | 7 |
| 56 | Peris | Spain | 2002 | 0 | 1 | 1 | 0 | 1 | 1 | 1 | 1 | 1 | 1 | 8 |
| 57 | Yamaga | Japan | 2000 | 1 | 1 | 1 | 1 | 1 | 1 | 0 | 1 |  |  | 7 |
| 58 | Gregorio | Argentina | 2000 | 1 | 1 | 1 | 1 | 1 | 1 | 0 | 1 |  |  | 7 |
| 59 | Anai | Japan | 1999 | 1 | 0 | 1 | 1 | 1 | 1 | 0 | 1 |  |  | 6 |
| 60 | Babbitt | USA | 1998 | 1 | 1 | 1 | 1 | 1 | 1 | 0 | 1 |  |  | 7 |
| 61 | Smith | England | 1995 | 0 | 0 | 1 | 0 | 0 | 1 | 1 | 1 | 1 | 1 | 7 |
| 62 | Yamamoto | Japan | 1994 | 1 | 0 | 1 | 1 | 1 | 1 | 0 | 1 |  |  | 6 |
| 63 | Rillo | Argentina | 1994 | 1 | 1 | 1 | 1 | 1 | 1 | 0 | 1 |  |  | 7 |
| 64 | Blanch | England | 1994 | 1 | 1 | 1 | 1 | 1 | 1 | 0 | 1 |  |  | 7 |
| 65 | Reid | New Zealand | 1992 | 1 | 1 | 1 | 1 | 1 | 1 | 1 | 1 |  |  | 8 |

^a^ The Joanna Briggs Institute (JBI) critical appraisal checklist for case reports and case series were employed to evaluate the quality of the included studies

Q question
